# STAT5 is activated in macrophages by breast cancer cell-derived factors and regulates macrophage function in the tumor microenvironment

**DOI:** 10.1186/s13058-021-01481-0

**Published:** 2021-11-07

**Authors:** Emily A. Jesser, Nicholas J. Brady, Danielle N. Huggins, Patrice M. Witschen, Christine H. O’Connor, Kathryn L. Schwertfeger

**Affiliations:** 1 Microbiology, Immunology and Cancer Biology Graduate Program, University of Minnesota, Minneapolis, MN USA; 2 Department of Laboratory Medicine and Pathology, 6Th St SE, University of Minnesota, Minneapolis, MN USA; 3 Comparative and Molecular Biosciences Graduate Program, University of Minnesota, Minneapolis, USA; 4 University of Minnesota Supercomputing Institute, University of Minnesota, Minneapolis, MN USA; 5 Masonic Cancer Center, University of Minnesota, Minneapolis, MN USA; 6 Center for Immunology, University of Minnesota, Minneapolis, USA; 7 Present Address: Department of Pathology and Laboratory Medicine, Weill Cornell Medicine, New York, NY 10021 USA

**Keywords:** Breast cancer, Tumor-associated macrophages, STAT5, Tumor microenvironment, Metastasis

## Abstract

**Background:**

In breast cancer, complex interactions between tumor cells and cells within the surrounding stroma, such as macrophages, are critical for tumor growth, progression, and therapeutic response. Recent studies have highlighted the complex nature and heterogeneous populations of macrophages associated with both tumor-promoting and tumor-inhibiting phenotypes. Defining the pathways that drive macrophage function is important for understanding their complex phenotypes within the tumor microenvironment. Signal transducer and activator of transcription (STAT) transcription factors, such as STAT5, are key regulators of immune cell function. The studies described here investigate the functional contributions of STAT5 to tumor-associated macrophage function in breast cancer.

**Methods:**

Initial studies were performed using a panel of human breast cancer and mouse mammary tumor cell lines to determine the ability of tumor cell-derived factors to induce STAT5 activation in macrophages. Further studies used these models to identify soluble factors that activate STAT5 in macrophages. To delineate STAT5-specific contributions to macrophage function, a conditional model of myeloid STAT5 deletion was used for in vitro*,* RNA-sequencing, and in vivo studies. The effects of STAT5 deletion in macrophages on tumor cell migration and metastasis were evaluated using in vitro co-culture migration assays and an in vivo tumor cell-macrophage co-injection model.

**Results:**

We demonstrate here that STAT5 is robustly activated in macrophages by tumor cell-derived factors and that GM-CSF is a key cytokine stimulating this pathway. The analysis of RNA-seq studies reveals that STAT5 promotes expression of immune stimulatory genes in macrophages and that loss of STAT5 in macrophages results in increased expression of tissue remodeling factors. Finally, we demonstrate that loss of STAT5 in macrophages promotes tumor cell migration in vitro and mammary tumor metastasis in vivo.

**Conclusions:**

Breast cancer cells produce soluble factors, such as GM-CSF, that activate the STAT5 pathway in macrophages and drive expression of inflammatory factors. STAT5 deletion in myeloid cells enhances metastasis, suggesting that STAT5 activation in tumor-associated macrophages protects against tumor progression. Understanding mechanisms that drive macrophage function in the tumor microenvironment will ultimately lead to new approaches that suppress tumor-promoting functions while enhancing their anti-tumor functions.

**Supplementary Information:**

The online version contains supplementary material available at 10.1186/s13058-021-01481-0.

## Introduction

Macrophages are a well-established component of the breast tumor microenvironment, and their roles in tumor growth and development are complex and multifaceted. In breast cancer, increased levels of infiltrating macrophages correlate with poor patient prognosis [1] as macrophages recruited to primary tumor and metastatic sites promote tumor cell survival, proliferation, therapeutic resistance, and evasion of the immune system [2–4]. However, macrophages also contribute to tumor elimination through enhancing adaptive immune responses by co-stimulation and antigen presentation [5, 6]. Tumor-associated macrophages (TAMs) produce soluble factors that interact with not only tumor cells, but also extracellular matrix (ECM) factors, vasculature components, and lymphocytes [3, 7] and contribute to the overall balance of a tumor-promoting or tumor-controlling microenvironment [8]. Studies characterizing TAM phenotypes in the tumor microenvironment have indicated TAM populations vary greatly based on tumor cell type, stage, localization, and stimuli [9, 10]. To effectively manipulate the balance between pro- and anti-tumor activity, it is important to understand the upstream mediators that regulate macrophage function in the breast cancer microenvironment.

The Janus Kinase (JAK)/Signal Transducer and Activator of Transcription (STAT) pathway is a critical regulator of macrophage function and we have previously shown that breast cancer-derived soluble factors are capable of activating both STAT3 and STAT5 in macrophages [11]. While STAT3 regulation of macrophages in the tumor microenvironment has been studied previously [11–14], there is significantly less known regarding the impact of STAT5 activation on TAMs. STAT5 is a well-known promoter of cell survival and its activation in mammary tumor cells has been linked to stimulation of oncogenic signaling pathways [15–20]. Some studies have demonstrated elevated pSTAT5 levels in early stages of tumor development, which is lost in more advanced stages of disease [16]. Other analyses have indicated STAT5 levels correlate with better outcomes in breast cancer patients [21, 22]. Together, these findings suggest that STAT5 is activated in epithelium early during the oncogenic process, and that loss of STAT5 activity is associated with late stages of tumor progression [16]. However, the effects of STAT5 activity in cells within the TME, such as in macrophages, have not yet been thoroughly addressed. Relevant to other immune cell types, STAT5 has a well-characterized role in T cell activation, survival, and lineage commitment [23–25] and STAT5 deletion in dendritic cells (DCs) results in impaired DC-stimulated T_H_2 responses [26]. Collectively, these findings indicate STAT5 regulates each arm of and intersections between innate and adaptive immunity. However, little is known about the contributions of STAT5 signaling to the function of TAMs, which can interact with both innate and adaptive immune cells. We have previously demonstrated loss of STAT5 in myeloid cells increases epithelial proliferation and hyperplasia formation in a mouse model of early stage tumorigenesis [27]. Together, these findings provided rationale to further evaluate STAT5-specific contributions to TAM function during breast cancer progression.

In these studies, we show that soluble factors, such as GM-CSF from triple-negative breast cancer (TNBC) cells, induce STAT5 activation in macrophages. Using genetic approaches, we demonstrate that STAT5 deletion via a *Csf1r-*driven Cre expression model enhances tumor cell metastasis to the lungs. Analysis of RNA-seq data reveals that loss of STAT5 in macrophages reduces expression of genes involved in immune stimulatory processes while increasing genes associated with tissue remodeling. Furthermore, we provide evidence that STAT5 deletion in macrophages can promote tumor cell migration in vitro and metastasis in vivo. Together, these studies suggest that the GM-CSF/STAT5 signaling axis restricts tumor-promoting functions of macrophages, and that loss of STAT5 activity in these cells results in a tumor-promoting microenvironment. Understanding the signaling mechanisms driving tumor-associated macrophage function is important for developing macrophage-focused therapeutic strategies for effective tumor control.

## Materials and methods

### Mice

*Csf1r-iCre* mice (Jackson Laboratories) and Stat5^fl/fl^ mice [generated by Dr. Lothar Hennighausen [28], obtained from Dr. Michael Farrar, University of Minnesota] were backcrossed to the BALB/c background, which was verified using congenic analysis (IDEXX-RADIL, Columbia, MO). STAT5^fl/fl^ and *Csf1r-iCre* mice were crossed to generate conditional knockout mice (STAT5^cKO^). Wild-type (WT) BALB/c mice were purchased from Envigo. All experiments were performed with 6- to 8-week-old female mice and all animal care and procedures were approved by the Institutional Animal Care and Use Committee of the University of Minnesota and in accordance with the procedures detailed in the Guide for the Care and Use of Laboratory Animals [29].

### Cell culture and stimulation

HC11 [30] and HC11/R1 cells were obtained from Jeffrey Rosen, Baylor College of Medicine, Houston, TX, and maintained as described previously [31]. To generate a cell line with enhanced take rate and metastatic propensity, HC11/R1 cells were injected into the mammary fat pad and primary tumors were harvested. Tumor cells were enriched for in culture by incubating in HC11 medium containing 2 mg/mL puromycin. Resulting cell lines were then injected into mammary fat pads of naïve mice and assessed for primary tumor formation and metastasis. The HC11/R1-LM cell line was identified based on its ability to metastasize to the lung following in vivo passage [32]. 4T1 cells were obtained from Thomas Griffith, University of Minnesota, Minneapolis, MN and grown in media containing RPMI, 10% FBS, 1% penicillin/streptomycin (Life Technologies), 1% L-glutamine (Life Technologies), 10 mM HEPES (Life Technologies), 1 mM sodium pyruvate (Life Technologies) 200 µg/mL G418. Human breast cancer and epithelial cell lines and THP-1 cells were obtained from and maintained in accordance with ATCC recommendations. Mouse BMDMs were maintained according to published protocols [33]. Human primary macrophages were derived from PBMCs isolated from Trima Cones obtained through the Memorial Blood Center, Minneapolis, MN. PBMCs were subjected to CD14 + enrichment via CD14 + microbeads (Miltenyi Biotec) through MACS LS columns (Miltenyi Biotec) and differentiated into macrophages with recombinant M-CSF (BioLegend) in RPMI supplemented with 10% FBS and 1% penicillin/streptomycin on days 1 and 5 (Day 5 treatment with 2X M-CSF) following CD14 + enrichment. All cells were grown at 37 °C and 5% CO2 and regularly checked for mycoplasma contamination. Serum-starved HC11, HC11/R1, and HC11/R1-LM cells were treated with 30 nM B/B (Clontech) or vehicle (ethanol) for 24 h, and conditioned media was collected, filtered, and used to stimulate BMDMs. 4T1 cells were serum-starved for conditioned media which was used to treat BMDMs. THP-1 cells were differentiated into macrophages with 5 ng/mL phorbol 12-myristate 13-acetate (PMA) overnight. Conditioned medium (CM) collected from serum-starved breast cancer cell lines was spun down to eliminate cellular debris and used to stimulate differentiated THP-1 or primary human macrophages that were serum-depleted in 1% FBS in DMEM for 4 h prior to CM exposure. In experiments neutralizing GM-CSF, rat anti-mouse GM-CSF (BioTechne) was incubated with tumor cell CM at a concentration of 2.5 µg/mL for 1 h at 37 °C prior to treatment of BMDMs. Normal mouse IgG (Santa Cruz) and no treatment were controls incubated with tumor cell CM prior to BMDM stimulation. BMDMs were stimulated with 4T1 CM for 24 h, media was replaced with fresh serum-free media for an additional 24 h to collect soluble factors from the STAT5^fl/fl^ and STAT5^cKO^ TAMs and referred to as STAT5^fl/fl^ DCM (double conditioned-media) and STAT5^cKO^ DCM, respectively. For non-contact co-culture experiments, 4T1 tumor cells were cultured in the bottom of a six-well plate along with STAT5^fl/fl^ or STAT5^cKO^ BMDMs plated in a 0.4 µm hanging insert to allow for soluble factor exchange.

### Migration assay

BMDMs were seeded at 2500 cells per 24-well 0.8 µm hanging insert in DMEM10 media and incubated overnight while 4T1 cells were starved in serum-free media. BMDMs in inserts were starved for 4 h prior to the addition of 1 × 10^4^ 4T1 cells. As controls, 4T1 cells were seeded alone. Six hundred microliters of RPMI-1640 medium containing 1% FBS was added to the lower chamber of the 24-well plate. After 20 h, cells on the apical side of the top chamber were removed with a cotton swab, inserts washed in PBS, then fixed with methanol for 10 min at − 20 °C. Cells which migrated to the lower side of the membrane were adhered to slide, coverslipped with DAPI and counted under a fluorescence microscope.

### Immunoblot analysis

Cells were lysed in RIPA buffer containing protease inhibitors and protein lysates were subjected to SDS–PAGE and immunoblot analysis as previously described [34]. Antibodies used include pSTAT5 (Cell Signaling #9314, 1:1000), total STAT5 (Cell Signaling #9363, 1:1000), β-tubulin (Cell Signaling # 2146S, 1:1000), pFAK Y397 (Cell Signaling, # 3283S, 1:1000), and total FAK (Cell Signaling #3285S, 1:1000).

### Quantitative RT-PCR

RNA for qRT-PCR was extracted from cells using TriPure trizol (Roche) and cDNA was prepared using the qScript cDNA synthesis kit (Quanta Biosciences) according to the manufacturers’ protocols. qRT-PCR was performed using PerfeCTa SYBR Green (Quanta Biosciences) and the Bio-Rad iQ5 system. The 2^−ΔΔCt^ method was used to determine relative quantification of gene expression and normalized to cyclophilin B (CYBP). Primer sequences: Human GMCSF: Fwd- CGTCTCCTGAACCTGAGTAGA, Rev- TGCTGCTTGTAGTGGCTG G. Mouse GMCSF: Fwd- GGCCTTGGAAGCATGTAGAGG, Rev- GGAGAACTCGTTAGAGACGACTT; Col1a1: Fwd– GACGCCATCAAGGTCTACTG, Rev- ACG GGA ATC CAT CGG TCA; Col5a2: Fwd- CAGAAGCCCAG ACGTATCG, Rev- GGTGGTCAGGCACTTCAGAT; Vegfa: Fwd- ACGTCAGAGAGCAACATCACC, Rev- CTTTGT TCT GTC TTT CTT TGG TCT G; Gpx1: Fwd- ATGTCGCGTCTCTCTGAGG, Rev- CCGAACTGATTGCACGGGAA; Bcl6: Fwd- CCGGCTCAATAATCTCGTGAA, Rev- GGTGCATGT AGAGTGGTGAGTGA.

### ELISA

Breast cancer cell CM samples were collected and used to perform a human GM-CSF DuoSet ELISA (R&D Systems). Murine tumor cell CM was used to perform mouse GM-CSF ELISA (R&D Systems). STAT5^fl/fl^ DCM and STAT5^cKO^ DCM was subjected to mouse Type I Collagen ELISA (Novus Biologicals). All ELISAs were performed according to the manufacturer’s instructions. 

### RNA-seq analysis

Total RNA was collected using TriPure reagent (Roche) from primary human STAT5^fl/fl^ and STAT5^cKO^ BMDMs treated with DMEM, 20 ng/mL rmGM-CSF (Fisher Scientific), or 4T1 CM. Samples were submitted in biological triplicate to the University of Minnesota Genomics Center for quality control, library creation, and next-generation sequencing. Due to quality control, one biological replicate was removed from the STAT5^fl/fl^ BMDM submissions. Sequencing data have been deposited in the gene expression omnibus (GEO) GSE171428.

### RNA-seq data processing

Bulk RNAseq samples were processed and aligned using the CHURP version 0.2.2 command line interface framework. A full description of the CHURP pipeline can be found in Baller et al. [35]. Briefly, trimmomatic version 0.33 was used to clean reads for adapter contamination and low-quality sequence, and FastQC was used to generate sequence quality reports for raw and trimmed reads [36]. HISAT2 version 2.1.0 was used to align samples to the genome reference consortium mouse build 38 reference genome [37]. featureCounts v1.6.2 was used to count mapped reads to genes [38]. M. musculus GRC build 38.99 gft file was used.

### Gene expression and pathway analysis

All differential gene expression and pathway analyses were done in R v 3.6.3 (R Core Team, 2020). Differential gene expression analysis was done in EdgeR v 3.28.1 [39]. Differentially expressed genes were identified between wild-type and Stat5 knockout samples for each treatment (DMEM, 4T1-CM and GM-CSF). Counts were normalized using the relative log expression normalization method and only genes with counts per million greater than one in two or more samples were kept. A general linear model approach was used to test for differentially expressed genes between wild-type and knockout samples for each treatment. A gene was categorized as differentially expressed if the p value was less than 0.01 after p value adjustment. P values were adjusted using the Benjamini & Hochberg method and there was no minimum log fold change required. GO term enrichment analysis and gene set enrichment analysis (GSEA) were done using the ClusterProfiler R package [40]. The hallmark gene set from the Molecular Signatures Database v 7.1 (https://www.gsea-msigdb.org/gsea/msigdb/index.jsp) was used for the GSEA analysis. Human gene orthologs of mouse genes were obtained from the Mouse Genome Informatics website (http://www.informatics.jax.org/downloads/reports/HMD_HumanPhenotype.rpt). 

### ELISA sample collection

Breast cancer cells were serum-starved for 24 h and conditioned medium samples were collected and spun at 1000 xg for 15 min at 4 °C. BSA in PBS was then added to each sample for a final concentration of 0.5%. Each sample was then transferred to a clean eppendorf tube and spun at 10,000 × g for 10 min at 4 °C, after which the supernatants were used for ELISA. Murine tumor cells were serum-starved for 24 h and conditioned media samples were collected and spun to eliminate cellular debris. BMDMs were stimulated with 4T1 CM for 24 h, media was replaced with fresh serum-free media for an additional 24 h to collect STAT5^fl/fl^ DCM and STAT5^cKO^ DCM.

### Microscope image acquisition

All images were taken on a Leica DM400B microscope at either 20 × or 40 × objectives. Images were acquired using a Leica DFC310 FX camera and LAS V3.8 software and processed in ImageJ. 3 images per lung were analyzed for metastasis.

### Tissue analysis

For immunofluorescence analysis, frozen OCT tumors were sectioned 5-μm-thick prior to staining. For paraffin embedded sections, tumors were fixed in 4% PFA and paraffin embedded. Lungs were inflated with 500µL of 2% PFA and fixed before paraffin embedding. Five-micrometer-thick sections were stained with hematoxylin and eosin (H&E).

### In vivo studies

For BALB/c mice tumor induction, 1 × 10^4^ HC11/R1-LM cells or 4T1 cells in 50% Matrigel (BD Biosciences) were injected into the inguinal mammary fat pads of 6-week-old mice. All mice receiving HC11/R1-LM tumors received 1 mg/kg B/B Homodimerizer (Clontech), intraperitoneally, twice weekly. For co-injections studies, BMDMs were treated with 4T1 CM for 24 h. CM-treated BMDMs were then harvested and injected at a ratio of 1:4 with 4T1 tumor cells. All mice were examined for tumor development by palpation and considered tumor-bearing once tumor size reached ∼100 mm^3^. Researchers were blinded to mouse genotype and co-injection group during data collection and analysis. Tumor volume was calculated using the following equation: V = (L × W2)/2. Mice were euthanized when tumors reached 1 cm^3^ and survival was recorded as number of days from surgery (Day 0) until tumor size endpoint.

### Tissue processing, macrophage isolation, and flow cytometry

At endpoint, lungs or tumors were harvested, minced, and digested in 1 mg/mL Collagenase D (Roche) containing 15 μg/mL DNaseI (Sigma-Aldrich) at 37 °C with shaking for 45–60 min. Following digestion, tissues were further homogenized through a 70 μm cell strainer and pelleted by centrifugation at 500 g. Red blood cells were lysed in ACK Buffer (150 mM ammonium chloride, 10 mM potassium chloride, 0.1 mM sodium EDTA, pH 7.4) and cells were resuspended in FACS Buffer (2% FBS and 1 mM EDTA in PBS). Macrophages were isolated using the Miltenyi F4/80 positive selection MACS beads (130-110-443) and lysed in RIPA buffer prior to immunoblot analysis. For flow cytometry analysis, cells were stained in an antibody master mix including fixable viability dye (eBioscience) and anti-CD16/CD32 (eBioscience, clone 93) at room temperature protected from light. Following surface antibody staining, samples were fixed in 2% paraformaldehyde (PFA) for 1 hour at room temperature. Cells were washed and permeabilized in 1 × Flow Cytometry Permeabilization Buffer (Tonbo) for 5 min, followed by incubation with intracellular antibodies in this buffer for 30 min at room temperature. Following a wash and centrifugation, cells were incubated with streptavidin-APC (eBioscience) for an additional 15 min at room temperature. Antibodies used include: CD45 (BD Biosciences, clone 30-F11), Ly6G (BioLegend, clone 1A8), CD64 (BioLegend, clone X54-5/7.1), MerTK (R&D Systems, polyclonal #BAF591, biotinylated). CountBright Absolute Counting Beads (Life Technologies) were used for cell number calculations. Samples were collected using a LSR Fortessa X-20 cytometer (BD Biosciences) and analyzed using FlowJo Software.

### Statistical analysis

Statistical analysis was performed using Student’s unpaired, two-tailed t test. Comparisons between at least three groups was performed using one-way ANOVA with Tukey’s multiple comparison test. Overall survival data were summarized using Kaplan–Meier curves and compared by treatment groups using log-rank tests (GraphPad PRISM v9). Error bars represent standard error of the mean (SEM). P values < 0.05 were considered statistically significant.

## Results

### Tumor cell-derived factors activate STAT5 in macrophages

**Fig. 1:**
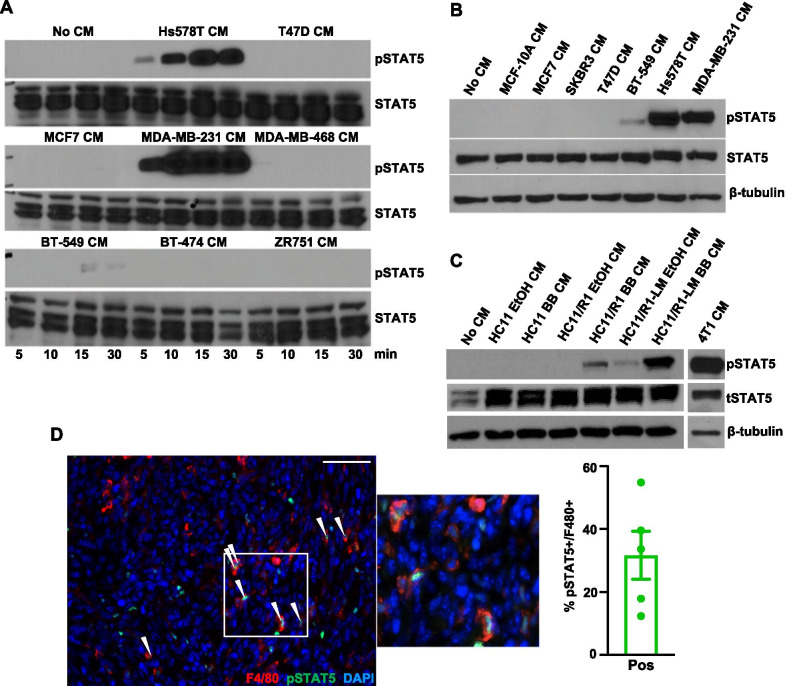
Tumor cell-derived soluble factors activate STAT5 signaling in macrophages. **A** Immunoblot for pSTAT5 and total STAT5 (STAT5) protein in THP-1 cells treated with the CM from the indicated human breast cancer cells for indicated times in minutes. **B** Immunoblot for pSTAT5, STAT5, and β-tubulin (loading control) protein in PBMC-derived macrophages treated with the CM from the indicated human breast cancer cells. **C** CM from B/B-stimulated HC11, HC11/R1, HC11/R1-LM and 4T1 cells was used to stimulate BMDMs. Immunoblot analysis for pSTAT5, STAT5, and β-tubulin. **D** 4T1 tumor sections were stained for F4/80 (red) and pSTAT5 (green). White arrows indicate co-stained cells. Scale bar: 50 μm. Quantification of percent of F4/80 + cells that were also pSTAT5 + from 5 representative tumors

JAK/STAT signaling is an important oncogenic pathway in both tumor and stromal cells [16, 17, 41–46]. While STAT1, STAT3, and STAT6 have been previously linked to TAM function [11, 12, 47], little is known regarding the function of STAT5 in TAMs. To determine whether tumor cells produce soluble factors that activate STAT5 in vitro, conditioned medium (CM) samples were collected from estrogen receptor positive (ER +) (T47D, MCF7, BT-474, ZR751), human epidermal growth factor receptor 2 (HER2 +) (BT-474, SKBR3), and TNBC (Hs578T, MDA-MB-231, MDA-MB-468, BT-549) cells. Soluble factors from serum-starved TNBC, but not ER + or HER2 + , cell lines induced STAT5 phosphorylation (pSTAT5) in THP-1 macrophages and peripheral blood mononuclear cell (PBMC)-derived macrophages (Fig. 1A, B). We also assessed the ability of murine mammary tumor cell lines to activate STAT5 in macrophages. 4T1 cells represent a well-characterized BALB/c-derived model of TNBC that efficiently metastasize to the lung following orthotopic injection [48]. To confirm these findings using an additional model, further studies were performed using a panel of cell lines derived from the immortalized, non-transformed HC11 mammary epithelial cell line [30]. We previously generated the HC11/R1 cell line, which is driven by an inducible FGFR1 oncogene when stimulated with a B/B homodimerizer and capable of tumor growth in vivo [34]. We have also recently generated a metastatic variant of these cells using in vivo passaging techniques, termed HC11/R1-LM [32]. Similar to the findings with human cell lines, soluble factors from serum-starved 4T1, B/B-stimulated HC11/R1, and B/B-stimulated HC11/R1-LM cells induced activation of STAT5 in mouse bone marrow derived macrophages (BMDMs) (Fig. 1C). Parental HC11 and solvent-stimulated HC11/R1 and HC11/R1-LM cells failed to activate STAT5, demonstrating that FGFR1 activation in epithelial cells leads to the production of soluble mediators that activate the STAT5 pathway in macrophages. To determine whether activation of STAT5 in macrophages occurs in mammary tumors in vivo, 4T1 cells were injected into the inguinal mammary fat pads of BALB/c mice and tumor sections were co-stained for pSTAT5 and the macrophage marker F4/80. Quantification determined that ~ 31% of F4/80 + cells are also pSTAT5 + (Fig. 1D). These results demonstrate soluble factors produced by human and murine tumor cells activate STAT5 in a subset of macrophages in the tumor microenvironment.

### Tumor cell-derived GM-CSF activates STAT5 in macrophages

**Fig. 2:**
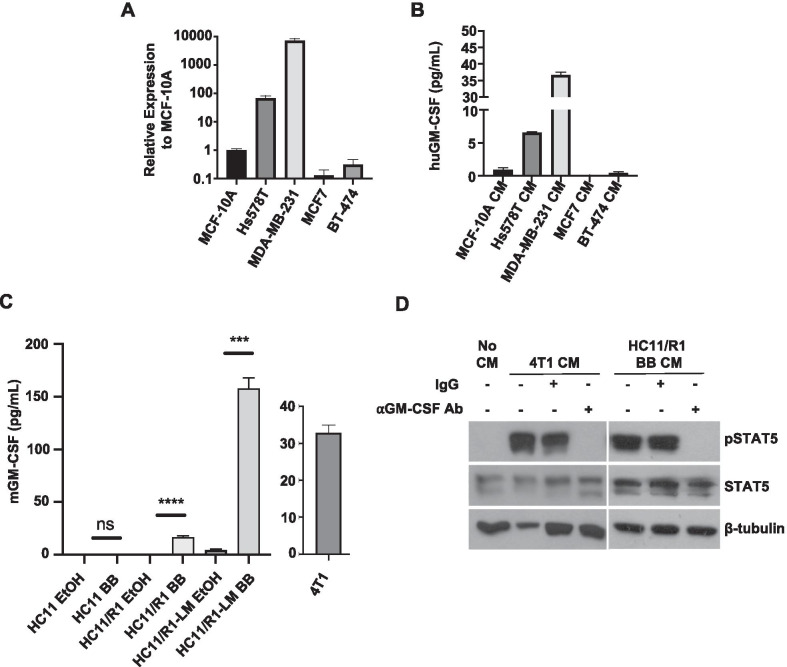
Tumor cell-derived GM-CSF activates STAT5 in macrophages. **A** qRT-PCR analysis for GM-CSF in TNBC (Hs578T and MDA-MB-231), ER + (MCF7) and HER2 + (BT-474) human breast cancer cells relative to expression in MCF-10A cells. **B** ELISA analysis for GM-CSF in CM collected from MCF-10A, MDA-MB-231, Hs578T, MCF7, and BT-474 cells. **C** ELISA analysis for GM-CSF in CM collected from 4T1 cells and B/B-stimulated HC11, HC11/R1, and HC11/R1-LM cells relative to EtOH controls. **D** Immunoblot analysis for pSTAT5, TSTAT5, and β-tubulin in BMDMs treated with No CM, tumor CM (4T1 or HC11/R1 BB), or tumor CM incubated for 1 h at 37 °C with 2.5 µg/mL neutralizing GM-CSF antibody (⍺GM-CSF Ab)

There are multiple avenues through which cell signaling leads to STAT5 activation. GM-CSF is a well-studied canonical activator of STAT5 signaling and is a cytokine that we and others have found to be produced by breast cancer cells [11, 49–51]. Therefore, we evaluated GM-CSF expression in a subset of the breast cancer cell lines used in Fig. 1. We found high GM-CSF expression in serum-starved Hs578T and MDA-MB-231 TNBC cells compared to MCF7 (ER +), BT-474 (HER2 +), and non-transformed epithelial MCF-10A cells. (Fig. 2A). Assessment of GM-CSF by ELISA revealed elevated concentrations in Hs578T and MDA-MB-231 CM, both of which induced pSTAT5 in macrophages (Fig. 2B). Analysis of GM-CSF production by the HC11, HC11/R1, and HC11/R1-LM cells demonstrated that B/B-treated HC11/R1 and HC11/R1-LM cells induced GM-CSF production (Fig. 2C). We and others have demonstrated that 4T1 cells produce soluble GM-CSF (Fig. 2C) [11, 52]. Further studies were performed to assess the functional contributions of GM-CSF to STAT5 activation in macrophages. Pre-treatment of 4T1-derived and HC11/R1-derived CM with a GM-CSF neutralizing antibody prior to BMDM stimulation led to a significant reduction in pSTAT5 in macrophages (Fig. 2D). These results suggest that tumor cell-derived GM-CSF contributes to STAT5 activation in macrophages.

### Myeloid deletion of STAT5 promotes metastasis

**Fig. 3:**
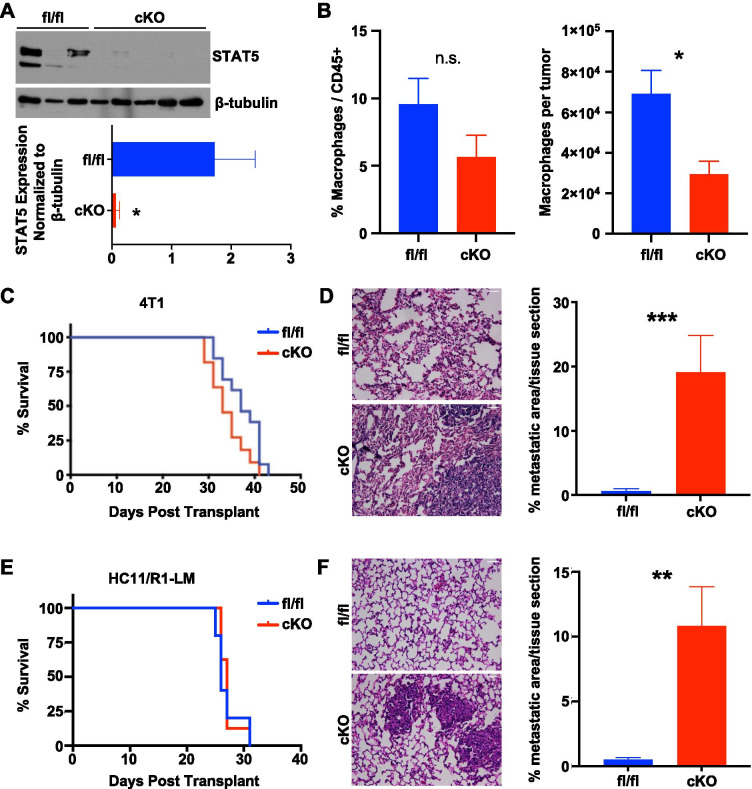
Deletion of STAT5 in myeloid cells enhances lung metastasis. **A** Immunoblot for total STAT5 in macrophages isolated from 4T1 tumors in fl/fl or cKO mice. Densitometry analysis relative to loading control. **B** Flow cytometric analysis of the relative frequency (among CD45 + cells) and total number of macrophages isolated from 4T1 tumors at endpoint. **C** Kaplan–Meier curves of 4T1 cells or **E** HC11/R1-LM cells transplanted into STAT5^fl/fl^ (n = 5–13) or STAT5^cKO^ (n = 8–11) mice. % Survival on Y-axes indicates proportion of mice reaching tumor size endpoint of 1cm^3^. **D** Lung sections from 4T1 and **F** HC11/R1-LM tumor-bearing mice stained for H&E and quantified percent metastatic area per tissue section. Lungs quantified from at least 4 mice per group. Unpaired *t-*test was used for statistical analysis. *P < 0.05, **P < 0.01. Scale bar: 50 μm

The immunocompetent 4T1 and HC11/R1 mammary tumor models were selected for further studies to assess the functional consequences of STAT5 activation in TAMs. STAT5-floxed mice were crossed with *Csfr1-iCre* mice to produce STAT5^fl/fl^ and STAT5^cKO^ BALB/c mice [27] and 1 × 10^4^ 4T1 tumor cells were injected into the inguinal mammary glands of the female mice. Isolation of macrophages from mammary tumors demonstrated a reduction in total STAT5 expression in macrophages from STAT5^cKO^ mice compared with STAT5^fl/fl^ mice (Fig. 3A). Further characterization of these mice demonstrated no significant differences in the frequency of macrophages within the CD45 + population, although there was a reduction in the total number of macrophages observed in tumors harvested from STAT5^cKO^ mice compared with STAT5^fl/fl^ mice (Fig. 3B). Further studies were performed to determine whether myeloid STAT5 deletion led to changes in mammary tumor growth and metastasis. While there were no significant differences observed in primary tumor growth between the two groups of mice (Fig. 3C), we observed a statistically significant increase in lung metastasis in the STAT5^cKO^ mice compared to the STAT5^fl/fl^ mice (Fig. 3D). These findings were confirmed using the HC11/R1-LM cells described above. 1 × 10^4^ tumor cells were injected into the mammary glands of STAT5^fl/fl^ and STAT5^cKO^ BALB/c mice. Similar to the 4T1 model, there were no significant differences in primary tumor onset, growth rate, or weight at endpoint between STAT5^fl/fl^ and STAT5^cKO^ tumor-bearing mice (Fig. 3E, Additional file 1: Fig. S1, S2). However, quantification of metastatic lesions in H&E-stained lungs demonstrated significantly increased metastasis in the STAT5^cKO^ mice (Fig. 3F). These data indicate that *Csfr1-*mediated deletion of STAT5 in the myeloid compartment results in enhanced lung metastasis, which suggests STAT5 activation in macrophages may be protective against metastatic progression.

### STAT5 in macrophages differentially regulates expression of adaptive immunity-related and tumor-promoting genes

**Fig. 4:**
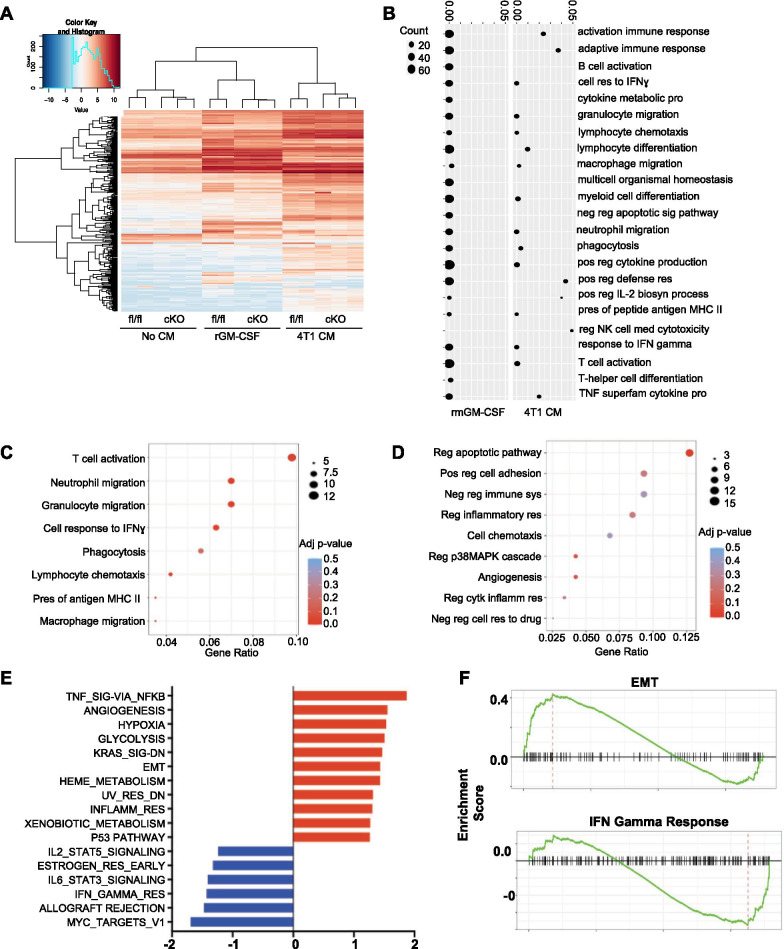
STAT5 in macrophages differentially regulates expression of adaptive immunity-related and tumor-promoting genes. **A** Heatmap of differentially regulated genes comparing STAT5^fl/fl^ or STAT5^cKO^ macrophages treated with No CM, recombinant GM-CSF (rmGM-CSF), or 4T1 CM. **B** Gene ontology analysis showing pathways altered based on genes that are downregulated in STAT5^cKO^ macrophages stimulated with rmGM-CSF (left panel) and 4T1 CM (right panel). **C**, **D** Gene ontology analysis showing biological processes downregulated and upregulated in 4T1 CM-treated STAT5^cKO^ BMDMs, respectively. **E** Positively enriched (red) and negatively enriched (blue) GSEA pathways in 4T1 CM-treated STAT5^cKO^ BMDMs. **F** The changes in expression of Epithelial to Mesenchymal Transition (top) and Interferon Gamma Response (bottom) genes in 4T1 CM-treated STAT5^cKO^ BMDMs analyzed by GSEA

To further explore the enhanced metastasis phenotype observed in STAT5^cKO^ mice, we sought to determine how STAT5 regulates gene expression in macrophages. STAT5^fl/fl^ and STAT5^cKO^ BMDMs were stimulated for 2 h with serum-free medium, recombinant mouse GM-CSF (rmGM-CSF), or 4T1 CM for RNA-seq analysis. Hierarchical clustering of the most variable genes revealed gene expression profiles vary between rmGM-CSF treatment and 4T1 CM. Secondary clustering occurs according to mouse genotype, suggesting differences in transcriptional behavior in stimulated BMDMs from the STAT5^cKO^ and STAT5^fl/fl^ mice (Fig. 4A). Gene ontology (GO) analysis determined that genes significantly reduced in rmGM-CSF STAT5^cKO^ macrophages were associated with recruitment, activation, and response of the adaptive immune system (Fig. 4B). To further investigate macrophage gene expression in the context of the tumor microenvironment, we focused on genes differentially expressed in STAT5^cKO^ BMDMS when stimulated with 4T1 CM. Similar to the rmGM-CSF treatment, downregulated genes in STAT5^cKO^ CM-treated macrophages resulted in adaptive immunity-related GO terms such as T cell activation, neutrophil, granulocyte and macrophage migration, and cellular response to IFNɣ (Fig. 4C). Interestingly, we also found upregulated genes in STAT5^cKO^ BMDMs associated with biological processes such as positive regulation of cell adhesion, negative regulation of immune system processes, regulation of the p38MAPK signaling cascade, and angiogenesis (Fig. 4D). Positive regulation of cell adhesion and angiogenesis processes are typically associated with tumor promotion and tissue remodeling, which can influence cell migration and extravasation events within the metastatic cascade [53]. Using gene set enrichment analysis (GSEA) to more globally identify patterns that otherwise may not be apparent, we found negative enrichment of the IL-2/STAT5 pathway in 4T1 CM-treated STAT5^cKO^ macrophages which confirms STAT5 activity is effectively diminished (Fig. 4E). GSEA revealed a positive enrichment of multiple cancer-associated pathways such as angiogenesis, hypoxia, glycolysis, and KRAS signaling in STAT5^cKO^ BMDMs (Fig. 4E). Notably, the epithelial to mesenchymal transition (EMT) gene set was also positively enriched in STAT5^cKO^ macrophages (Fig. 4F). EMT is critical during the early steps of the metastatic cascade and genes expressed in macrophages associated with this pathway include many ECM factors and genes known to contribute to tissue remodeling, such as *Col1a1, Col5a2, Lox, Mmp14, Vcan, Lamc1, and Bgn* [54]. In contrast, the IFNɣ response gene set was negatively enriched in STAT5^cKO^ BMDMs (Fig. 4F) and includes genes involved in co-stimulation (*Cd86)*, leukocyte recruitment (*Ccl2, Ccl7, Cxcl10)*, antigen presentation (*Cd74, Psme1, Ciita)*, and cell growth control (*Pim1*) [55, 56]. These findings suggest that STAT5 is important for regulating genes associated with adaptive immune responses and that loss of STAT5 leads to increased expression of tumor-promoting genes.

**Fig. 5:**
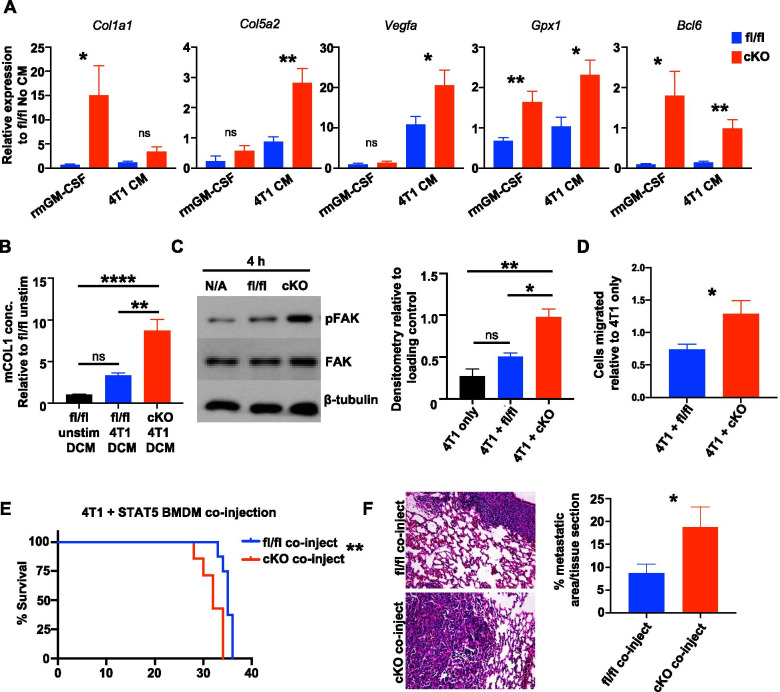
STAT5 deletion in macrophages enhances tumor-promoting phenotype and impacts tumor cell migration and metastasis. **A** qRT-PCR analysis of genes of interest from RNA-seq associated with tumor-promoting pathways in rmGM-CSF or 4T1 CM-treated STAT5^fl/fl^ (blue) and STAT5^cKO^ (red) BMDMs. Unpaired t-test was used for statistical analysis. **B** Mouse Type 1 Collagen ELISA in STAT5^fl/fl^ unstimulated or 4T1 CM-stimulated STAT5^fl/fl^ and STAT5^cKO^ macrophage double CM (DCM). Data were analyzed using one-way ANOVA and Tukey’s multiple comparison test. **C** Representative immunoblot of pFAK, total FAK (FAK), and β-tubulin in 4T1 cells cultured alone or co-cultured with STAT5^fl/fl^ or STAT5^cKO^ BMDMs for 4 h. Densitometry analysis relative to loading control. **D** Migration analysis of 4T1 cells cultured alone or co-cultured with STAT5^fl/fl^ or STAT5^cKO^ BMDMs after 20 h. Cell counts relative to 4T1 alone in triplicate. **E** Kaplan–Meier curves of 4T1 cells co-injected with either STAT5^fl/fl^ (n = 8) or STAT5^cKO^ (n = 7) BMDMs in WT BALB/c mice. % Survival on Y-axes indicates proportion of mice reaching tumor size endpoint of 1cm^3^. **F** Quantified metastasis in H&E-stained lung sections. Lungs were sectioned at 3 different depths per mouse and analyzed for percent metastatic area per tissue section. *P < 0.05, **P < 0.01, ***P < 0.001, ****P < 0.0001. Scale bar: 50 μm

Further studies were performed to validate key genes of interest that are associated with a tumor-promoting phenotype. Collagens are important components of the ECM and are found in abundance in invasive breast cancers [57, 58]. Studies have linked high levels of collagen crosslinking with stromal stiffness and found these tissues harbored the highest number of TAMs and found collagen-expressing TAMs in recurrent tumors [59–61]. Additional studies have found stromal-produced collagen augments the adhesion capacity of triple-negative MDA-MB-231 cells [62, 63]. Collagen genes were enriched in the EMT gene set from our GSEA analysis, therefore we validated gene expression in rmGM-CSF and 4T1 CM-treated BMDMs from STAT5^fl/fl^ and STAT5^cKO^ mice. We found *Col1a1* and *Col5a2* were significantly upregulated in STAT5^cKO^ macrophages (Fig. 5A). We also evaluated collagen production by macrophages at the protein level. STAT5^cKO^ and STAT5^fl/fl^ BMDMs were stimulated with 4T1 CM for 24 h, then CM was removed and replaced with serum-free media to collect the macrophage-produced soluble factors in what we refer to as double conditioned media (DCM). Using DCM, we performed an ELISA and found that STAT5^cKO^ BMDMs produced significantly higher concentrations of type I collagen than STAT5^fl/fl^ macrophages (Fig. 5B). Furthermore, we analyzed STAT5^cKO^ macrophages for expression of additional tumor-promoting genes including *Vegfa*, *Gpx1,* and *Bcl6*. *Vegfa* expression stimulates blood vessel formation, which can aid in supplying growth factors and nutrients to support tumor growth and metastasis [50, 64]. *Gpx1* (glutathione peroxidase 1) encodes for a cytosolic antioxidant enzyme important for modulating reactive oxygen species in the tumor microenvironment that is elevated in M2-polarized TAMs in breast cancer [65, 66]. Finally, *Bcl6* is a gene involved in cell adhesion, negative regulation of the immune response, and inflammation [67, 68] and myeloid-specific deficiency of Bcl6 has been shown to decrease tumor growth and metastasis [69]. These genes were all found to be significantly upregulated in STAT5^cKO^ BMDMs (Fig. 5A). These results indicate STAT5^cKO^ macrophages express and produce higher levels of ECM factors and other factors associated with TAM function than STAT5^fl/fl^ macrophages.

### STAT5 deletion in macrophages enhances tumor cell migration and metastasis

Our data indicate that loss of STAT5 in macrophages results in increased expression of tumor-promoting factors. Macrophage-mediated changes in ECM composition or tissue remodeling factors can drive tumor cell migration, therefore, we hypothesized that STAT5^cKO^ macrophages directly promote tumor cell metastatic properties such as migration. Focal adhesion kinase (FAK) is a well-known contributor to tumor cell adhesion and invasiveness, and as it is overexpressed in many metastatic tumors [70], it is an attractive anticancer target [66, 71]. The RNA-seq analysis indicates that deletion of STAT5 in macrophages results in increased expression of factors known to activate FAK signaling, such as growth factors (*Egf, Vegfa, Ccn2),* collagens*,* and glycoproteins *(Tnc)* [70, 72]*.* To discern effects on FAK in tumor cells, we cultured BMDMs in transwells with tumor cells in the lower chamber. This non-contact co-culture system allows for the assessment of changes in tumor cells as a result of soluble factor exchange with STAT5^fl/fl^ or STAT5^cKO^ macrophages. 4T1 cells co-cultured with STAT5^cKO^ BMDMs displayed increased levels of pFAK when compared to 4T1 cells alone or co-cultured with STAT5^fl/fl^ BMDMs via immunoblotting (Fig. 5C). Because FAK activation is associated with tumor cell migration, further studies were performed to determine whether STAT5^cKO^ macrophages promote migration. In a transwell migration assay, we observed significantly enhanced tumor cell migration when 4T1 cells were co-cultured with STAT5^cKO^ macrophages compared to STAT5^fl/fl^ co-culture (Fig. 5D). These results suggest loss of STAT5 leads to a tumor-promoting expression profile and generates macrophages capable of influencing tumor cell migration.

As an alternative approach to the *Csfr1-iCre* mice, in which Cre-mediated gene deletion is not limited specifically to TAMs [73], we determined whether co-injection of tumor cells with pre-conditioned macrophages lacking STAT5 would be sufficient to enhance tumor cell aggressiveness and metastatic potential. In this experiment, BMDMs isolated from STAT5^fl/fl^ and STAT5^cKO^ mice were pre-treated with 4T1 CM and then co-injected with 1 × 10^4^ tumor cells at a ratio of 1:4. Primary tumor growth and lung metastasis were assessed as described above. 4T1 cells co-injected with STAT5^cKO^ macrophages reached tumor size endpoint faster, demonstrating enhanced primary tumor growth compared with 4T1 cells co-injected with STAT5^fl/fl^ macrophages (Fig. 5E). Consistent with our hypothesis, we also found significantly enhanced lung metastasis in mice co-injected with 4T1 tumor cells and STAT5^cKO^ TAMs (Fig. 5F). Taken together, these studies indicate that loss of STAT5 expression in TAMs leads to enhanced tumor metastasis.

## Discussion

Macrophages are highly infiltrative within breast tumors and are known to influence disease outcomes [1]. Conventionally, macrophages are characterized as M1 (classically activated) or M2 (alternatively activated). M1- and M2-polarized macrophages are commonly identified by expression of specific markers based on the stimulus in the microenvironment. M1 macrophages are proinflammatory and considered antitumor and M2 macrophages are associated with antagonizing inflammation and promoting wound healing [74]. TAMs are often categorized as M2 but recent studies have suggested TAMs can express markers associated with both polarization states depending on tumor type and stage, suggesting a spectra of macrophage phenotypes found in the tumor microenvironment [9, 10]. As these cells have the capacity to behave in either a tumor-promoting or tumor-antagonizing manner, it is important to determine the upstream events that dictate their function. STAT5 has been linked to both M1 and M2 macrophage polarization in different models [75–80], suggesting that STAT5 function in macrophages may be context dependent. We demonstrate here that loss of STAT5 signaling in macrophages enhances metastasis by promoting tumor cell migration and contributing to the formation of a more permissive environment to disease progression in mammary tumors. These studies further indicate that the GM-CSF/STAT5 signaling axis may tip the balance of macrophage activity towards an anti-tumor immune response and contribute to tumor control.

We demonstrate here that TNBC cell-derived GM-CSF activates STAT5 in macrophages. It is important to note that the conditioned media for these experiments were collected under serum free conditions in order to reduce potential non-specific effects of serum on signaling pathways in macrophages. The increased levels of GM-CSF production by TNBC cell lines compared with other cell line subtypes suggest higher constitutive levels of inflammatory signaling pathways in TNBC cells in the absence of exogenous stimulation, which has been observed previously [81]. However, these findings do not address the possibility that ER+ and HER2+ cells can be induced to secrete GM-CSF with exogenous factors. For example, GM-CSF can also be produced by MCF7 cells induced to undergo EMT following TGFβ stimulation [81]. Thus, while published data support a higher level of GM-CSF expression in basal breast cancer samples [50], further studies are needed to identify the specific signaling pathways regulating GM-CSF expression in TNBC cells, and to determine whether exogenous factors such as serum, hormones, or growth factors induce or enhance production of GM-CSF in all subtypes of breast cancer cells.

The role of GM-CSF in breast cancer is unclear as evidence suggests GM-CSF can support tumor growth but also exhibits inhibitory effects [82, 83]. The function of GM-CSF in various immune cell populations in the tumor microenvironment has been extensively studied, as this cytokine is vital for survival and differentiation of DCs and monocytes/macrophages [49, 82]. Studies have implicated cancer cell-derived GM-CSF in promoting disease progression and immune suppression (i.e. supporting myeloid-derived suppressor cells or MDSCs) [51, 84–86]. Other studies have determined GM-CSF stimulates antitumor immunity through activation and antigen presentation of DCs, as well as priming of T cells [87, 88]. Recent studies have begun to further investigate the effects of tumor-derived GM-CSF on TAMs in breast cancer as they have not been previously extensively characterized [52, 81, 89]. For example, 4T1-derived GM-CSF has been shown to promote an M1 phenotype in macrophages and exhibit anti-metastatic function [52]. While GM-CSF can initiate signaling through multiple pathways including STAT5, MAPK, and PI3K/Akt [90], the distinct pathways leading to changes in macrophage phenotype have not been specifically investigated. Our data suggest GM-CSF/STAT5 signaling in macrophages has a critical function in regulating tumor/stroma interactions in breast cancer.

While STAT5 activity is known to promote survival and oncogenic signaling in mammary epithelial and breast cancer cells [15, 16, 21, 22], less is known regarding how STAT5 regulates macrophage function. We previously demonstrated that pSTAT5 is activated in approximately 30% of macrophages in proximity to developing terminal end buds [27]. However, these studies did not include assessment of STAT5 phosphorylation in macrophage populations within the adipose stroma, which, as we have recently described, represents a large proportion of resident macrophages in the mammary gland [91]. We demonstrate here that STAT5 is activated in approximately 31% of tumor-associated macrophages. Further studies using approaches that allow for spatial resolution of expression levels of STAT5 activating cytokines, such as GM-CSF, would provide additional insight into the mechanisms driving the heterogeneity of STAT5 activation in the macrophage population in both the normal mammary gland and in tumors. Considering the robust STAT5 activation in macrophages by tumor-derived factors, we sought to determine whether STAT5 deletion impacted mammary tumor progression. To this end, we generated a mouse model in which STAT5 deletion is driven in myeloid lineages by *Csf1r-iCre*. In previously published studies using this model, we demonstrated that myeloid STAT5 deletion leads to altered mammary gland development [27]. Specifically, we found a reduction in ductal elongation along with increased branching and epithelial proliferation in the mammary gland. Further analysis of the mammary glands from these mice demonstrated no detectable defects in the recruitment of macrophages to the epithelial ducts. In contrast, here we identified a reduction in the number of macrophages recruited to mammary tumors in the STAT5^cKO^ mice, suggesting potential implications for STAT5 in regulating tumor-associated macrophage differentiation, survival, proliferation, or recruitment of bone marrow derived and/or resident macrophages. Given that STAT5 is a potent survival factor in mammary epithelial cells [15–17], it would be of interest to assess whether it contributes similarly to the survival of TAMs in this model.

We demonstrate here that STAT5 deletion using the *Csf1r-iCre* model led to enhanced tumor cell metastasis to the lung. STAT5 activation has been associated with M1 macrophage polarization [92] which suggests this transcription factor may have the capacity to promote anti-tumor immune responses in macrophages. RNA-seq analysis of STAT5^fl/fl^ and STAT5^cKO^ macrophages revealed genes significantly reduced in STAT5^cKO^ BMDMs were associated with anti-tumor and adaptive immune responses such as T cell activation and recruitment, as well as antigen presentation and phagocytosis. Conversely, genes significantly increased with STAT5 deletion were associated with pro-tumor, tissue remodeling/repair biological processes. Consistent with the results from the *Csf1r-iCre* model in vivo*,* enhanced metastasis in the STAT5^cKO^ mice may be attributed to macrophage-mediated suppression of the adaptive immune response and coinciding tumor microenvironment alterations promoting migration and invasion. Further exploration of STAT5-mediated changes in macrophages revealed positive enrichment of gene sets associated with TNF signaling via NFκB, angiogenesis, and EMT, among others. Additionally, these studies focused primarily on GM-CSF as an activator of STAT5 in macrophages; whether other STAT5-activating stimuli induce similar patterns of transcriptional regulation in macrophages remains to be examined.

In STAT5^cKO^ macrophages, we validated increased expression of a subset of genes related to EMT and angiogenesis and also demonstrated the ability of these cells to produce increased levels of collagen in vitro. Fibroblasts are major contributors to the synthesis of ECM components such as collagen [62] and TAMs are key drivers of tissue remodeling, partially due to their expression of matrix-metalloproteinases and other factors responsible for ECM degradation [93]. Macrophages have also been previously studied for their ability to instruct fibroblast production of collagens [63] but here, we show macrophage-mediated collagen secretion in 4T1 CM-treated STAT5^cKO^ BMDMs. Notably, these cells increased expression of multiple types of collagen genes known to promote tumor growth and metastasis (Type I, II, IV, and V collagens) [2, 59, 63]. Tumor cells are responsive to changes in ECM molecules through integrin mediated FAK activation. FAK activation is known to control cell migration and invasion [70] and as a result, FAK inhibitors are currently being evaluated for their therapeutic efficacy in reducing tumor growth and metastasis in breast cancer [71]. Using non-contact co-culture methods, we demonstrated that the STAT5^cKO^ macrophages produce soluble factors that induce FAK activation and promote tumor cell migration. Soluble growth factors, glycoproteins, and collagens are capable of activating FAK [70] and further studies are required to define the specific factors produced by the STAT5^cKO^ macrophages that activate FAK in the tumor cells. It would be interesting to also assess FAK activity in a direct cell-to-cell contact system as this would also be relevant in modeling the TME. In addition to tumor/stroma interactions, it would also be useful to evaluate how STAT5 in macrophages influences endothelial cells and T cells since STAT5^cKO^ BMDMs highly expressed angiogenesis- and adaptive immune response-related genes.

Cre expression in the *Csf1r-iCre* model is found in myeloid cells and a subset of splenic lymphocytes [73]. Additionally, myeloid deletion of STAT5 also impacts cells within both the primary tumor and the pre-metastatic niche [94]. Therefore, we used an additional in vivo approach in order to more specifically determine how STAT5-deficient macrophages in the primary tumor influence tumor growth and metastasis. In WT BALB/c mice, we co-injected 4T1 tumor cells with either CM-stimulated STAT5^fl/fl^ or STAT5^cKO^ BMDMs. Interestingly, we observed a significant difference in primary tumor growth with the STAT5^cKO^ co-injected group reaching tumor size end point sooner. This result may be due to the pre-conditioning of the macrophages prior to injection with tumor cells, allowing for a more rapid disease progression than tumor cells alone. Consistent with our hypothesis, we observed significantly enhanced lung metastasis in STAT5^cKO^ co-injected mice, suggesting that STAT5^cKO^ macrophages can function within the primary tumor to enhance tumor growth rate and the ability of the tumor cells to metastasize.

Due to its oncogenic potential, the JAK/STAT pathway is an attractive therapeutic target especially for TNBC patients who otherwise have very limited treatment options. However, this pathway is also important to non-tumor cells, such as TAMs. While previous studies have assessed STAT5 levels and phosphorylation in human breast cancers, STAT5 activation has not been specifically assessed in tumor-associated macrophages in human samples. Active STAT5 in tumor cells is generally associated with a more favorable prognosis in human breast cancer patients and loss of STAT5 is associated with the acquisition of a malignant phenotype [16, 21, 22]. While these studies did not directly address STAT5 levels in macrophages, it would be interesting to determine whether this is associated with a concomitant decrease in STAT5 activation in macrophages and a reduction in tumor restraining properties of these macrophages. The inclusion of techniques that allow for transcriptomic and proteomic spatial resolution to assess changes in STAT5-activating cytokines in these regions would also contribute to our understanding of STAT5 activation in tumor cells and the microenvironment. It is necessary to understand the functional contributions of STATs in TAMs, as these cells may impact therapeutic efficacy. To this end, we have demonstrated STAT5 in macrophages protects against metastatic progression and disruption of this signaling in macrophages enhances the malignant potential of tumor cells. We also identified GM-CSF as an important upstream contributor to macrophage STAT5 activation, which provides rationale to further explore methods of selectively targeting this signaling to enhance macrophage immuno-stimulatory potential without inducing tumor-promoting effects in other cell types. One such method could be the application of cell-specific GM-CSF cytokine delivery to macrophages in the tumor microenvironment [95]. Obtaining a better understanding of the mechanisms through which macrophages impact tumor progression will ultimately lead to the development of approaches that exploit their potential anti-tumorigenic properties for therapeutic purposes.

## Conclusions

In summary, we demonstrate that mammary tumor-derived GM-CSF is an important cytokine involved in the activation of STAT5 signaling in macrophages. Further investigation by RNA-sequencing analysis revealed STAT5 regulates genes associated with the anti-tumor immune response in macrophages. We also found that loss of STAT5 in macrophages increased their expression of tumor-promoting factors and enhanced tumor cell migration and metastasis in vitro and in *vivo*, respectively. These studies provide rationale for further exploration of the GM-CSF/STAT5 signaling axis in harnessing the anti-tumor potential of tumor-associated macrophages in the mammary tumor microenvironment.

## Supplementary Information

**Additional file 1.** Supplemental Figures S1 and S2.

## Data Availability

The datasets generated during and/or analyzed during the current study are available in gene expression omnibus (GEO) GSE171428.

## Abbreviations

GM-CSF: Granulocyte–macrophage colony-stimulating facto
STAT: Signal transducer and activator of transcription
TAM: Tumor-associated macrophage
ECM: Extracellular matrix
JAK: Janus kinase
DC: Dendritic cell
TNBC: Triple-negative breast cancer
CM: Conditioned medium/media
ER: Estrogen receptor
HER2: Human epidermal growth factor receptor
PBMC: Peripheral blood mononuclear cell
BMDM: Bone marrow-derived macrophage
GO: Gene ontology
GSEA: Gene set enrichment analysis
DCM: Double-conditioned media

## References

[1] Bingle L, Brown NJ, Lewis CE (2002). The role of tumour-associated macrophages in tumour progression: implications for new anticancer therapies. J Pathol.

[2] Pollard JW (2008). Macrophages define the invasive microenvironment in breast cancer. J Leukoc Biol.

[3] Noy R, Pollard JW (2014). Tumor-associated macrophages: from mechanisms to therapy. Immunity.

[4] Williams CB, Yeh ES, Soloff AC (2016). Tumor-associated macrophages: unwitting accomplices in breast cancer malignancy. Breast Cancer.

[5] Mosser DM, Edwards JP (2008). Exploring the full spectrum of macrophage activation. Nat Rev Immunol.

[6] Mills CD, Lenz LL, Harris RA (2016). A breakthrough: macrophage-directed cancer immunotherapy. Cancer Res.

[7] Biswas SK, Mantovani A (2010). Macrophage plasticity and interaction with lymphocyte subsets: cancer as a paradigm. Nat Immunol.

[8] Clément AU, Fernando TA, Paola A (2019). Current strategies to targettumor-associated-macrophages to improveanti-tumor immune responses. Cells.

[9] Cassetta L, Fragkogianni S, Sims AH, Swierczak A, Forrester LM, Zhang H (2019). Human tumor-associated macrophage and monocyte transcriptional landscapes reveal cancer-specific reprogramming, biomarkers, and therapeutic targets. Cancer Cell.

[10] Wagner J, Rapsomaniki MA, Chevrier S, Anzeneder T, Langwieder C, Dykgers A (2019). A single-cell atlas of the tumor and immune ecosystem of human breast cancer. Cell.

[11] Irey EA, Lassiter CM, Brady NJ, Chuntova P, Wang Y, Knutson TP (2019). JAK/STAT inhibition in macrophages promotes therapeutic resistance by inducing expression of protumorigenic factors. Proc Natl Acad Sci.

[12] Wang T, Niu G, Kortylewski M, Burdelya L, Shain K, Zhang S (2004). Regulation of the innate and adaptive immune responses by Stat-3 signaling in tumor cells. Nat Med.

[13] Zhou J, Qu Z, Sun F, Han L, Li L, Yan S (2017). Myeloid STAT3 promotes lung tumorigenesis by transforming tumor immunosurveillance into tumor-promoting inflammation. Cancer Immunol Res.

[14] Kujawski M, Kortylewski M, Lee H, Herrmann A, Kay H, Yu H (2008). Stat3 mediates myeloid cell-dependent tumor angiogenesis in mice. J Clin Invest.

[15] Rädler PD, Wehde BL, Wagner KU (2017). Crosstalk between STAT5 activation and PI3K/AKT functions in normal and transformed mammary epithelial cells. Mol Cell Endocrinol.

[16] Wagner KU, Rui H (2008). Jak2/Stat5 signaling in mammogenesis, breast cancer initiation and progression. J Mammary Gland Biol Neoplasia.

[17] Schmidt JW, Wehde BL, Sakamoto K, Triplett AA, Anderson SM, Tsichlis PN (2014). Stat5 regulates the phosphatidylinositol 3-kinase/Akt1 pathway during mammary gland development and tumorigenesis. Mol Cell Biol.

[18] Arendt LM, Rugowski DE, Grafwallner-Huseth TA, Garcia-Barchino MJ, Rui H, Schuler LA (2011). Prolactin-induced mouse mammary carcinomas model estrogen resistant luminal breast cancer. Breast Cancer Res.

[19] Johnston AN, Bu W, Hein S, Garcia S, Camacho L, Xue L (2018). Hyperprolactinemia-inducing antipsychotics increase breast cancer risk by activating JAK-STAT5 in precancerous lesions. Breast Cancer Res.

[20] Haricharan S, Dong J, Hein S, Reddy JP, Du Z, Toneff M (2013). Mechanism and preclinical prevention of increased breast cancer risk caused by pregnancy. Elife.

[21] Peck AR, Witkiewicz AK, Liu C, Stringer GA, Klimowicz AC, Pequignot E (2011). Loss of nuclear localized and tyrosine phosphorylated Stat5 in breast cancer predicts poor clinical outcome and increased risk of antiestrogen therapy failure. J Clin Oncol.

[22] Peck AR, Witkiewicz AK, Liu C, Klimowicz AC, Stringer GA, Pequignot E (2012). Low levels of Stat5a protein in breast cancer are associated with tumor progression and unfavorable clinical outcomes. Breast Cancer Res.

[23] Yao Z, Cui Y, Watford WT, Bream JH, Yamaoka K, Hissong BD (2006). Stat5a/b are essential for normal lymphoid development and differentiation. Proc Natl Acad Sci USA.

[24] Tripathi P, Kurtulus S, Wojciechowski S, Sholl A, Hoebe K, Morris SC (2010). STAT5 is critical to maintain effector CD8 + T cell responses. J Immunol.

[25] Park JH, Adoro S, Guinter T, Erman B, Alag AS, Catalfamo M (2010). Signaling by intrathymic cytokines, not T cell antigen receptors, specifies CD8 lineage choice and promotes the differentiation of cytotoxic-lineage T cells. Nat Immunol.

[26] Bell BD, Kitajima M, Larson RP, Stoklasek TA, Dang K, Sakamoto K (2013). STAT5 is critical in dendritic cells for development of Th2- but not Th1- dependent immunity. Nat Immunol.

[27] Brady NJ, Farrar MA, Schwertfeger KL (2017). STAT5 deletion in macrophages alters ductal elongation and branching during mammary gland development. Dev Biol.

[28] Cui Y, Riedlinger G, Miyoshi K, Tang W, Li C, Deng C-X (2004). Inactivation of Stat5 in mouse mammary epithelium during pregnancy reveals distinct functions in cell proliferation, survival, and differentiation. Mol Cell Biol.

[29] Animals NRC (US) C for the U of the G for the, Laboratory C and U of. Guide for the Care and Use of Laboratory Animals. Guid Care Use Lab Anim. 2011.

[30] Ball RK, Friis RR, Schoenenberger CA, Doppler W, Groner B (1988). Prolactin regulation of beta-casein gene expression and of a cytosolic 120-kd protein in a cloned mouse mammary epithelial cell line. EMBO J.

[31] Xian W, Schwertfeger KL, Vargo-Gogola T, Rosen JM (2005). Pleiotropic effects of FGFR1 on cell proliferation, survival, and migration in a 3D mammary epithelial cell model. J Cell Biol.

[32] DN H, RS L, Y W, TP K, Y X, JW W, et al. Characterizing macrophage diversity in metastasis-bearing lungs reveals a lipid-associated macrophage subset. Cancer Res. 2021;canres.0101.2021. 10.1158/0008-5472.CAN-21-0101 PMC8530952 34389631

[33] Freedman TS, Tan YX, Skrzypczynska KM, Manz BN, Sjaastad F V., Goodridge HS, et al. LynA regulates an inflammation-sensitive signaling checkpoint in macrophages. Elife. 2015;4(OCTOBER2015). 10.7554/eLife.09183 PMC4626889 26517880

[34] Bohrer LR, Chuntova P, Bade LK, Beadnell T, Leon RP, Brady NJ (2014). Activation of the FGFR-STAT3 pathway in breast cancer cells induces a hyaluronan-rich microenvironment that licenses tumor formation. Cancer Res.

[35] Baller J, Kono T, Herman A, Zhang Y (2019). ChURP: a lightweight CLI framework to enable novice users to analyze sequencing datasets in parallel. Proc Pract Exp Adv Res Comput Rise Mach.

[36] Bolger AM, Lohse M, Usadel B (2014). Trimmomatic: a flexible trimmer for Illumina sequence data. Bioinformatics.

[37] Kim D, Paggi JM, Park C, Bennett C, Salzberg SL (2019). Graph-based genome alignment and genotyping with HISAT2 and HISAT-genotype. Nat Biotechnol.

[38] Liao Y, Smyth GK, Shi W (2014). FeatureCounts: an efficient general purpose program for assigning sequence reads to genomic features. Bioinformatics.

[39] Robinson MD, McCarthy DJ, Smyth GK (2009). edgeR: a bioconductor package for differential expression analysis of digital gene expression data. Bioinformatics.

[40] Yu G, Wang LG, Han Y, He QY (2012). ClusterProfiler: an R package for comparing biological themes among gene clusters. Omi A J Integr Biol.

[41] Yu H, Jove R (2004). The STATs of cancer–new molecular targets come of age. Nat Rev Cancer.

[42] Balko JM, Schwarz LJ, Luo N, Estrada M V, Giltnane JM, Dávila-González D, et al. Triple-negative breast cancers with amplification of JAK2 at the 9p24 locus demonstrate JAK2-specific dependence. Sci Transl Med. 2016;8(334):334ra53. 10.1126/scitranslmed.aad3001 PMC5256931 27075627

[43] Marotta LLC, Almendro V, Marusyk A, Shipitsin M, Schemme J, Walker SR, et al. The JAK2 / STAT3 signaling pathway is required for growth of CD44 + CD24 – stem cell – like breast cancer cells in human tumors. 2011;121(7):2723–35. 10.1172/JCI44745 PMC3223826 21633165

[44] Iavnilovitch E, Cardiff RD, Groner B, Barash I (2004). Deregulation of Stat5 expression and activation causes mammary tumors in transgenic mice. Int J Cancer.

[45] Creamer BA, Sakamoto K, Schmidt JW, Triplett AA, Moriggl R, Wagner K-U (2010). Stat5 Promotes Survival of Mammary Epithelial Cells through Transcriptional Activation of a Distinct Promoter in Akt1. Mol Cell Biol.

[46] O’Shea JJ, Holland SM, Staudt LM (2013). JAKs and STATs in immunity, immunodeficiency, and cancer. N Engl J Med.

[47] Binnemars-Postma K, Bansal R, Storm G, Prakash J (2018). Targeting the Stat6 pathway in tumor-associated macrophages reduces tumor growth and metastatic niche formation in breast cancer. FASEB J.

[48] Aslakson CJ, Miller FR. Selective events in the metastatic process defined by analysis of the sequential dissemination of subpopulations of a mouse mammary tumor. Cancer Res. 1992;52(6). 1540948

[49] Lehtonen A, Matikainen S, Miettinen M, Julkunen I (2002). Granulocyte-macrophage colony-stimulating factor (GM-CSF)-induced STAT5 activation and target-gene expression during human monocyte/macrophage differentiation. J Leukoc Biol.

[50] Fertig EJ, Lee E, Pandey NB, Popel AS (2015). Analysis of gene expression of secreted factors associated with breast cancer metastases in breast cancer subtypes. Sci Rep.

[51] Ghirelli C, Reyal F, Jeanmougin M, Zollinger R, Sirven P, Michea P (2015). Breast cancer cell-derived GM-CSF licenses regulatory Th2 induction by plasmacytoid predendritic cells in aggressive disease subtypes. Cancer Res.

[52] Brenot A, Knolhoff BL, Denardo DG, Longmore GD (2018). SNAIL1 action in tumor cells influences macrophage polarization and metastasis in breast cancer through altered GM-CSF secretion. Oncogenesis.

[53] Valastyan S, Weinberg RA (2011). Tumor metastasis: molecular insights and evolving paradigms. Cell.

[54] Merl-Pham J, Basak T, Knüppel L, Ramanujam D, Athanason M, Behr J, et al. Quantitative proteomic profiling of extracellular matrix and site-specific collagen post-translational modifications in an in vitro model of lung fibrosis. Matrix Biol Plus. 2019;1:100005. 10.1016/j.mbplus.2019.04.002 PMC7852317 33543004

[55] Zhang S, Kim CC, Batra S, McKerrow JH, Loke P. Delineation of diverse macrophage activation programs in response to intracellular parasites and cytokines. PLoS Negl Trop Dis. 2010;4(3). 10.1371/journal.pntd.0000648 PMC2846935 20361029

[56] Ong S-M, Tan Y-C, Beretta O, Jiang D, Yeap W-H, Tai JJY (2012). Macrophages in human colorectal cancer are pro-inflammatory and prime T cells towards an anti-tumour type-1 inflammatory response. Eur J Immunol.

[57] Natal R de A, Paiva GR, Pelegati VB, Marenco L, Alvarenga CA, Vargas RF, et al. Exploring collagen parameters in pure special types of invasive breast cancer. Sci Rep. 2019;9(1):1–11. 10.1038/s41598-019-44156-9 PMC6531485 31118443

[58] Acerbi I, Cassereau L, Dean I, Shi Q, Au A, Park C (2015). Human breast cancer invasion and aggression correlates with ECM stiffening and immune cell infiltration. Integr Biol (United Kingdom).

[59] Maller O, Drain AP, Barrett AS, Borgquist S, Ruffell B, Zakharevich I, et al. Tumour-associated macrophages drive stromal cell-dependent collagen crosslinking and stiffening to promote breast cancer aggression. Nat Mater. 10.1038/s41563-020-00849-5 PMC8005404 33257795

[60] Provenzano PP, Inman DR, Eliceiri KW, Knittel JG, Yan L, Rueden CT, et al. Collagen density promotes mammary tumor initiation and progression. 2008; 10.1186/1741-7015-6-11 PMC2386807 18442412

[61] Walens A, Dimarco AV, Lupo R, Kroger BR, Damrauer JS, Alvarez JV (2019). CCL5 promotes breast cancer recurrence through macrophage recruitment in residual tumors. Elife.

[62] Kim SH, Lee HY, Jung SP, Kim S, Lee JE, Nam SJ (2014). Role of secreted type I collagen derived from stromal cells in two breast cancer cell lines. Oncol Lett.

[63] Afik R, Zigmond E, Vugman M, Klepfish M, Shimshoni E, Pasmanik-Chor M (2016). Tumor macrophages are pivotal constructors of tumor collagenous matrix. J Exp Med.

[64] Boudreau N, Myers C (2003). Breast cancer-induced angiogenesis: multiple mechanisms and the role of the microenvironment. Breast Cancer Res.

[65] Griess B, Mir S, Datta K, Teoh-Fitzgerald M (2020). Scavenging reactive oxygen species selectively inhibits M2 macrophage polarization and their pro-tumorigenic function in part, via Stat3 suppression. Free Radic Biol Med.

[66] Lee E, Choi A, Jun Y, Kim N, Yook JI, Kim SY (2020). Glutathione peroxidase-1 regulates adhesion and metastasis of triple-negative breast cancer cells via FAK signaling. Redox Biol.

[67] Walker SR, Nelson EA, Zou L, Chaudhury M, Signoretti S, Richardson A (2009). Reciprocal effects of STAT5 and STAT3 in breast cancer. Mol Cancer Res.

[68] Bos R, Van Diest PJ, Van Der Groep P, Greijer AE, Hermsen MAJA, Heijnen I (2003). Protein expression of B-cell lymphoma gene 6 (BCL-6) in invasive breast cancer is associated with cyclin D1 and hypoxia-inducible factor-1α (HIF-1α). Oncogene.

[69] Desai NN, ZHU B, Son Y, Sun J. Inhibition of effective anti-tumor immunity by macrophage Bcl6. J Immunol. 2019;202(1 Supplement).

[70] Yoon H, Dehart JP, Murphy JM, Lim STS (2015). Understanding the roles of FAK in cancer: inhibitors, genetic models, and new insights. J Histochem Cytochem.

[71] Jean C, Chen XL, Nam JO, Tancioni I, Uryu S, Lawson C (2014). Inhibition of endothelial FAK activity prevents tumor metastasis by enhancing barrier function. J Cell Biol.

[72] Sieg DJ, Hauck CR, Ilic D, Klingbeil CK, Schaefer E, Damsky CH (2000). FAK integrates growth-factor and integrin signals to promote cell migration. Nat Cell Biol.

[73] McCubbrey AL, Allison KC, Lee-Sherick AB, Jakubzick C V., Janssen WJ. Promoter specificity and efficacy in conditional and inducible transgenic targeting of lung macrophages. Front Immunol. 2017 Nov 24;8(NOV):24. 10.3389/fimmu.2017.01618 PMC5705560 29225599

[74] Martinez FO, Gordon S. The M1 and M2 paradigm of macrophage activation: time for reassessment. F1000Prime Rep. 2014;6. 10.12703/P6-13 PMC3944738 24669294

[75] Huen SC, Huynh L, Marlier A, Lee Y, Moeckel GW, Cantley LG (2015). GM-CSF promotes macrophage alternative activation after renal ischemia/reperfusion injury. J Am Soc Nephrol.

[76] Zhang Y, Li X, Luo Z, Ma L, Zhu S, Wang Z (2020). ECM1 is an essential factor for the determination of M1 macrophage polarization in IBD in response to LPS stimulation. Proc Natl Acad Sci USA.

[77] Wang N, Liang H, Zen K. Molecular mechanisms that influence the macrophage M1-M2 polarization balance. Front Immunol. 2014;5(NOV). 10.3389/fimmu.2014.00614 PMC4246889 25506346

[78] Qin S, Li J, Zhou C, Privratsky B, Schettler J, Deng X (2020). SHIP-1 regulates phagocytosis and M2 polarization through the PI3K/Akt–STAT5–Trib1 circuit in pseudomonas aeruginosa Infection. Front Immunol.

[79] Kuroda E, Ho V, Ruschmann J, Antignano F, Hamilton M, Rauh MJ (2009). SHIP represses the generation of IL-3-induced M2 macrophages by Inhibiting IL-4 production from basophils. J Immunol.

[80] Li J, Li C, Zhuang Q, Peng B, Zhu Y, Ye Q, et al. The evolving roles of macrophages in organ transplantation. J Immunol Res. 2019;2019. 10.1155/2019/5763430 PMC6507224 31179346

[81] Su S, Liu Q, Chen J, Chen J, Chen F, He C (2014). A Positive feedback loop between mesenchymal-like cancer cells and macrophages is essential to breast cancer metastasis. Cancer Cell.

[82] Hong I-S. Stimulatory versus suppressive effects of GM-CSF on tumor progression in multiple cancer types. Exp Mol Med. 2016;242. 10.1038/emm.2016.64 PMC4973317 27364892

[83] Eubank TD, Roberts RD, Khan M, Curry JM, Nuovo GJ, Kuppusamy P (2009). Granulocyte macrophage colony-stimulating factor inhibits breast cancer growth and metastasis by invoking an anti-angiogenic program in tumor-educated macrophages. Cancer Res.

[84] Ravindranathan S, Nguyen KG, Kurtz SL, Frazier HN, Smith SG, Koppolu BP (2018). Tumor-derived granulocyte colony-stimulating factor diminishes efficacy of breast tumor cell vaccines. Breast Cancer Res.

[85] Morales JK, Kmieciak M, Knutson KL, Bear HD, Manjili MH (2010). GM-CSF is one of the main breast tumor-derived soluble factors involved in the differentiation of CD11b−Gr1− bone marrow progenitor cells into myeloid-derived suppressor cells. Breast Cancer Res Treat.

[86] Bayne LJ, Beatty GL, Jhala N, Clark CE, Rhim AD, Stanger BZ (2012). Tumor-derived granulocyte-macrophage colony-stimulating factor regulates myeloid inflammation and T cell immunity in pancreatic cancer. Cancer Cell.

[87] Gupta R, Emens LA (2010). GM-CSF-secreting vaccines for solid tumors: moving forward. Discov Med.

[88] Dranoff G (2002). GM-CSF-based cancer vaccines. Immunol Rev.

[89] Sami E, Paul BT, Koziol JA, El Shamy WM (2020). The immunosuppressive microenvironment in BRCA1-IRIS-overexpressing TNBC tumors is induced by bidirectional interaction with tumor-associated macrophages. Cancer Res.

[90] Lotfi N, Thome R, Rezaei N, Zhang GX, Rezaei A, Rostami A, et al. Roles of GM-CSF in the pathogenesis of autoimmune diseases: an update. Front Immunol. 2019 Jun 4;10(JUN):1265. 10.3389/fimmu.2019.01265 PMC6593264 31275302

[91] Wang Y, Chaffee TS, LaRue RS, Huggins DN, Witschen PM, Ibrahim AM (2020). Tissue-resident macrophages promote extracellular matrix homeostasis in the mammary gland stroma of nulliparous mice. Elife.

[92] Martinez FO, Gordon S. The M1 and M2 paradigm of macrophage activation: time for reassessment. F1000Prime Rep. 2014;6(13):1–13. 10.12703/P6-13 PMC3944738 24669294

[93] Kessenbrock K, Plaks V, Werb Z (2010). Matrix metalloproteinases: regulators of the tumor microenvironment. Cell.

[94] Eddy WE, Gong K-Q, Bell B, Parks WC, Ziegler SF, Manicone AM (2017). Stat5 is required for CD103 + dendritic cell and alveolar macrophage development and protection from lung injury. J Immunol.

[95] Ve Garcin G, Paul F, Staufenbiel M, Bordat Y, Van Der Heyden J, Wilmes S, et al. High efficiency cell-specific targeting of cytokine activity. Nat Commun. 2014;5(3016). 10.1038/ncomms4016 24398568

