# Supplementary material for: STAT5 is activated in macrophages by breast cancer cell-derived factors and regulates macrophage function in the tumor microenvironment
Source: Breast Cancer Res. 2021 Nov 7;23:104. doi: 10.1186/s13058-021-01481-0 (PMC8573892; doi:10.1186/s13058-021-01481-0)
Supplement: Supplementary file 1 — Additional file 1. Supplemental Figures S1 and S2. [file 13058_2021_1481_MOESM1_ESM.pdf]

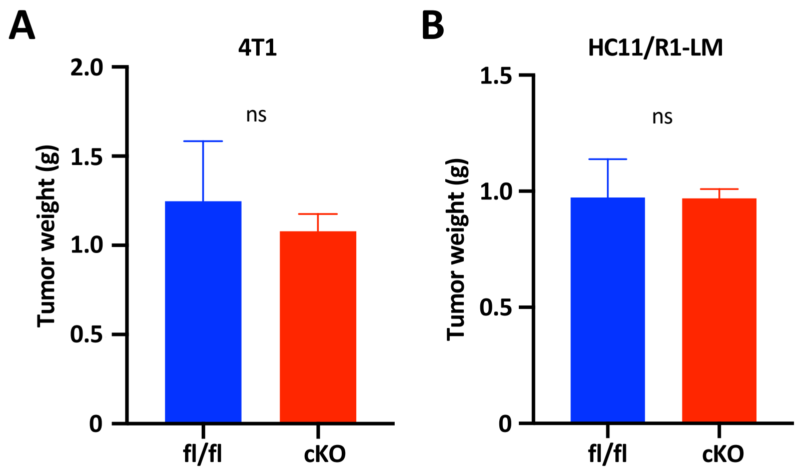

Supplemental Figure 1. Tumor weight at size endpoint does not differ between $STAT5^{fl/fl}$ and $STAT5^{cKO}$ mice. A Tumor weights of 4T1 tumors and **B** HC11/R1-LM tumors transplanted into $STAT5^{fl/fl}$ (n = 5-13) or $STAT5^{cKO}$ (n = 8-11) mice. % Survival on Y-axes indicates proportion of mice reaching tumor size endpoint of 1cm3, not disease-related death.

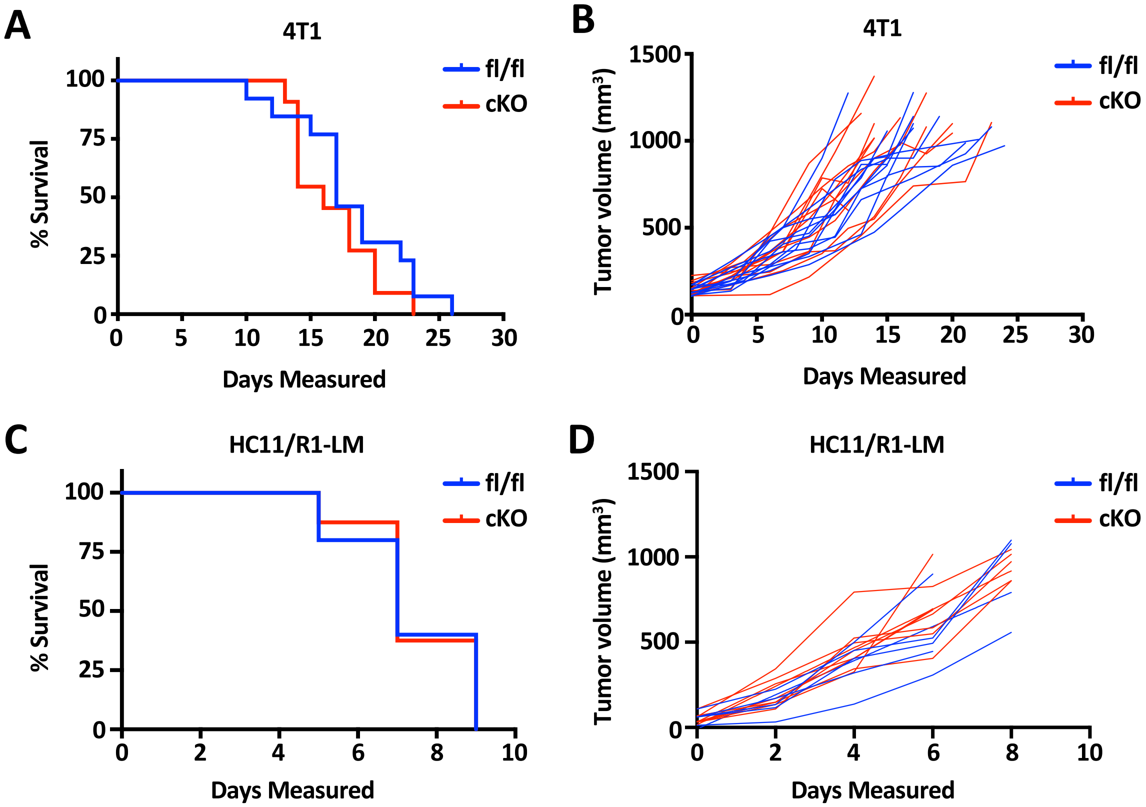

Supplemental Figure 2. Deletion of STAT5 in myeloid cells does not impact overall tumor growth rate. A Kaplan–Meier curves of 4T1 or **C** HC11/R1-LM tumors transplanted into $STAT5^{fl/fl}$ (n = 5-13) or $STAT5^{cKO}$ (n = 8-11) mice. % Survival on Y-axes indicates proportion of mice reaching tumor size endpoint of $1cm^3$ (Day 0 = palpable tumor), not disease-related death. **B** Individual tumor growth rates from 4T1 and **D** HC11/R1-LM tumor-bearing mice (Day 0 = palpable tumor).
